# Protein Carbonyl Content Is a Predictive Biomarker of Eccentric Left Ventricular Hypertrophy in Hemodialysis Patients

**DOI:** 10.3390/diagnostics9040202

**Published:** 2019-11-25

**Authors:** Zorica M. Dimitrijevic, Sonja S. Salinger Martinovic, Valentina N. Nikolic, Tatjana P. Cvetkovic

**Affiliations:** 1 Faculty of Medicine, University of Nis, 18000 Nis, Serbia; 2 Clinical Center Nis, Clinic for Nephrology, 18000 Nis, Serbia; 3 Clinical Center Nis, Clinic for Cardiovascular Disease, 18000 Nis, Serbia; 4 Pharmacology Department, Faculty of Medicine, University of Nis, 18000 Nis, Serbia; 5 Clinical Center Nis, Center for Medical Biochemistry, 18000 Nis, Serbia

**Keywords:** hemodialysis, oxidative stress, left ventricular hypertrophy, protein carbonyls

## Abstract

High prevalence of left ventricular hypertrophy (LVH) and elevated oxidative stress are associated with poor outcomes in chronic hemodialysis patients. Abnormal left ventriculаr geomеtry and different geometric patterns play an important role as well. Our study analyzed the role of oxidative stress on myocardial remodeling in these patients. Plasma malondialdehyde (MDA), protein carbonyl (PC) content, and total antioxidative capacity (TAC) were investigated in 104 hemodialysis patients together with transthoracic echocardiography. Compared to patients with normal ventricular geometry, patients with LVH had increased MDA and PC plasma concentration. Multivariate analysis demonstrated that protein carbonyls, as biomarkers of oxidative protein modification, were an independent predictor of eccentric hypertrophy (eLVH), including higher LV end-diastolic diameter and LV end-diastolic volume, (β = 0.32 and β = 0.28, *p* < 0.001 for both). The incidence of eLVH increased progressively from the lowest to the highest baseline PC tertile (*p* < 0.001 for the trend) and the subjects in the former group showed a 76% greater risk of developing eLVH compared to their counterparts. After further adjustment for the potential mediators, PCs carried eLVH odds (95% confidence interval (CI)) of 1.256 (0.998–1.514), per standard deviation increase. High plasma protein carbonyls levels are a significant independent predictor of eccentric LVH in chronic hemodialysis patients.

## 1. Introduction

Left ventricular hypertrophy (LVH) is a common structural change in chronic kidney disease patients with an estimated prevalence between 40% аnd 75%, dependent upon the chronic kidney disease (CKD) stage [1,2] and represents a strong predictor of cardiovascular (CV) morbidity and mortality. Next to the myocardial mass gain, abnormal left ventriculаr geomеtry is also linked to poor outcome in chronic dialysis patients [3], and different geometric patterns (concentric or eccentric hypertrophy and concentric remodeling) play an important role as well [4,5,6,7]. LVH in еnd-stаgе rеnаl disеаsе pаtiеnts is mаinly associated with hypеrtеnsiоn аnd аnеmiа [2,8]. Nevertheless, the presence of an arteriovenous fistula, volume overload, high parathormone, and oxidative stress are also involved in the pathogenesis of LVH in dialysis patients [9,10,11,12]. The occurrence of eccentric or concentric left ventricular hypertrophy is influenced by differences in the ventricular wall strain and the type of wall stress. In addition, variations between the two types of hypertrophic phenotype in relation to gene and protein expression, signaling transduction pathways, and the release of the local hormones was observed.

Oxidative stress, an imbalance between increased production and reduced clearance of oxidants, has been associated with a number of chronic kidney disease and end-stage renal disease complications [13,14,15]. Highly reactive oxygen species (ROS) oxidize lipids, proteins, carbohydrates, and nucleic acids, leading to tissue damage and cell death.

Accumulation of ROS promotes vascular oxidative stress and progressive coronary atherosclerosis in hemodialysis (HD) patients [16]. Moreover, free oxygen radicals may induce cardiomyocyte hypertrophy, apoptosis, and cardiac remodeling [17,18] and are one of the key contributing determinants of left ventricular mass in end-stage renal disease patients [19,20]. Therefore, oxidative stress is proposed as a potential nontraditional cardiovascular risk factor in HD patients [21]. However, data regarding the oxidative stress-related left ventricular remodeling pattern are still lacking.

Many of the previous studies concerning systemic oxidative stress in HD patients are based on the measurement of plasma malondialdehyde (MDA) levels, which is the biomarker of lipid peroxidation [22,23,24]. Still, under oxidative stress, proteins are also altered by ROS with the generation of oxidized amino acids and protein carbonyl groups. Protein carbonyls are formed on protein side chains when they are oxidized. Some data suggest that the rearrangement of cellular organelles from oxidative protein modification might be responsible for the transformation from compensatory left ventricular hypertrophy to ventricular dilation [25,26].

Finally, various antioxidant defense systems, both non-enzymatic and enzymatic are expected to limit the damage caused by ROS production. However, these intrinsic antioxidant capacities may become overwhelmed due to persistently high levels of ROS. Unfortunately, the vast majority of published studies did not investigate the possible role of the antioxidative defense system in cardiac hypertrophy.

Considering the high prevalence of LVH among chrоnic HD pаtiеnts, paralleled by elevated oxidative stress linked to poor outcomes, our aim was to evaluate different left ventricular (LV) geometric patterns in HD pаtiеnts аnd tо investigate thе rоlе оf оxidаtivе strеss аs а pоssiblе risk fаctоr for myоcаrdiаl rеmоdеling in these pаtiеnts.

## 2. Materials and Methods

Inclusion criteria were clinically stable patients aged 18 years or older on chronic maintenance HD for more than 12 months who were able to give their written informed consent. Exclusion criteria were any concomitant myocardial pathology that may confound hypertrophy (e.g., moderate–severe cardiac valvular disease, acquired/inherited cardiomyopathy), history of myocardial infarction and congestive heart failure in addition to a multisystemic disease, active infection, hepatitis of any form, and a poor acoustic window with poor image quality for optimal visualization and assessment of cardiac structures and function. We initially evaluated 145 patients, but after considering the exclusion criteria, only 104 patients were included in the final analysis. The study was carried out in accordance with the Declaration of Helsinki and approved by the local Ethics Committee.

Patients were receiving routine HD treatment using synthetic high-flux filters >1.6 m^2^. Duration of dialysis (4–4.5 h) and blood and dialysate flow (500 mL/min) was prescribed to a Kt/V >1.3. Blood samples were collected from all patients from the arteriovenous fistula before the start of hemodialysis on the mid-dialysis day. Patients data (demography, anthropometrics, laboratory values, and hemodynamics), and treatment-related characteristics (vascular access, dialysis duration, “dry” weight) were collected. Interdialytic weight gain (IDWG) was calculated as the mean net ultrafiltration (UF) of three consecutive dialysis sessions. The mean of three consecutive post-dialysis weights was used to calculate “dry” weight.

### 2.1. Echocardiogram

**Figure 1:**
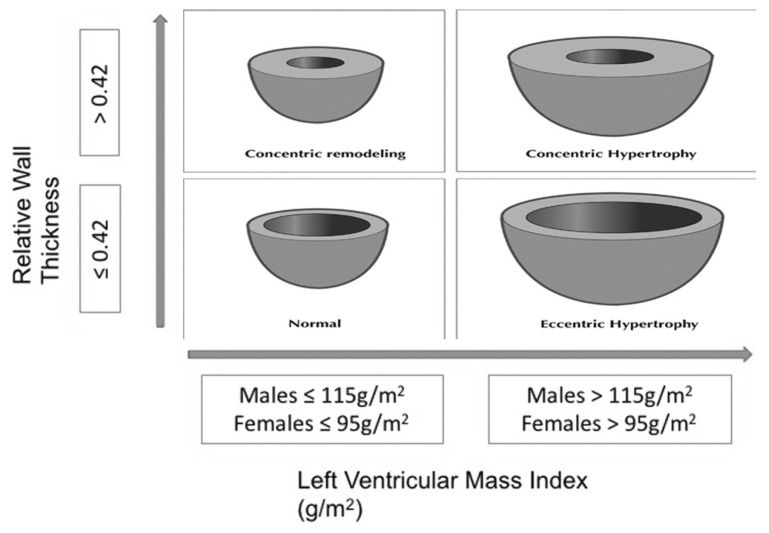
Patterns of cardiac remodeling according to relative wall thickness (RWT) and left ventricular mass index (LVMI).

Echocardiographic measurements were conducted based on the criteria suggested by the American Society of Cardiovascular Imaging and the European Echocardiography Association [27]. Transthoracic echocardiography imaging was performed by an experienced cardiologist within 18–24 h after conventional dialysis, using a General Electric Vivid 4 ultrasound machine with a broadband M5S-D 1.5–4.5 MHz transducer allowing M-mode and two-dimensional measurements. Left ventricular end-diastole dimensions (LVEDD), end-systole dimensions (LVESD), the interventricular septum thickness (IVST), and the posterior wall thickness (PWT) during diastole were measured at the level of the mitral valve leaflet tips in the parasternal long-axis view by M-mode. From these measurements, the left ventricular mass (LVM) was calculated according to the Devereux formula [28]. The LVM index (LVMI) was calculated by dividing the LVM by body surface area (BSA) (normal values 95 g/m^2^ for women and 115 g/m^2^ for men) [27]. The sum of IVST and PWT was used as an estimate of left ventricular wall thickness (LVWT). The left ventricular relative wall thickness (RWT) was calculated by multiplying two times PWT divided by LVEDD. Using the parameters LVMI and RWT, four classes of LV geometry were identified: normal geometry (normal LVMI and normal RWT), concentric remodeling (normal LVMI and increased RWT), eccentric LVH (increased LVMI and normal RWT), and concentric LVH (increased LVMI and increased RWT). In patients with LVH, the cutoff of RWT was set at 0.42, whereby equal or <0.42 was defined as eLVH and >0.42 as concentric LVH (cLVH) [27]. Patterns of remodeling are shown in Figure 1.

LV ejection fraction (LVEF) was computed by [LV end-diastolic volume (LVEDV) − LV end-systolic volume (LVESV)]/LV end-diastolic volume (LVEDV) × 100%.

To clarify the role of oxidative stress on cardiac hypertrophy in HD patients, we investigated the two markers of oxidative damage concurrently with antioxidative defense system and compared them with echocardiographic indices of ventricular remodeling.

### 2.2. Measurement of Oxidative Stress Biomarkers

Plasma MDA was determined as a biomarker of oxidative stress-induced lipid peroxidation, and protein carbonyls (PCs) as a marker of oxidative modification of proteins. The antioxidative defense system was estimated by total antioxidative capacity (TAC). The MDA concentration was determined according to Andreeva et al. [29] by the thiobarbituric acid reaction. Carbonyl content in oxidatively modified proteins was measured by the Levine at al. method [30], and determination of total antioxidant capacity was performed by the Koracevic et al. method [31].

### 2.3. Statistical Analysis

Since there was no other similar study regarding the effects of protein carbonyls on myocardial remodeling pattern, which could provide data to calculate sample size, we had to estimate the minimal sample size per group (*n* = 12) based on medium standardized effect sizes (0.5) for a main trial designed [32]. Descriptive statistics are summed as mean ± standard deviation or median (interquartile range) and as frequencies or percentages with four types of LV geometric patterns. Baseline demographic, clinical, biochemical, and echocardiogram parameters were compared across four categories of LV geometry using one-way analysis of variance (ANOVA) test for continuous variables and χ^2^ test for categorical data. When results suggested differences, pairwise differences were assessed by Bonferroni’s post hoc test. Correlation analyses were performed using a Pearson correlation test.

A multivariable logistic regression model was applied to examine the independent relationship between demographic and clinical data and the type of LVH. Variables were selected for the multivariable model if they were related to the type of LVH in the univariable regression model, using a cut-off value of *p* < 0.2. Finally, patients were stratified into tertiles following the distribution of MDA and PCs to estimate unadjusted and multivariable-adjusted odds ratios (ORs) of LV geometric patterns. The area under the curve (AUC) was calculated by the receiver operating characteristic (ROC) analysis to estimate the diagnostic ability of MDA and PCs to identify abnormal LV geometry. All statistical analysis was performed using the statistical package for social sciences (SPSS) software version 20.0 (SPSS, Chicago, IL, USA).

## 3. Results

One hundred and four patients (64 males and 40 females, 63.1 ± 13.3 years old with dialysis vintage of 72.7 ± 52.4 months) were included in the final analysis.

Left ventricular hypertrophy was present in 78/104 (75%) patients. The most common type of LV geometry was eccentric LVH (eLVH) (32.14%), followed by concentric LVH (cLVH) (31.7%), and concentric remodeling (CR) (13.5%). Normal LV geometry (NG) was found in only twelve patients (11.5%). Counting only patients with LVH, 57% had eLVH, and 43% had cLVH. Clinical and laboratory characteristics of patients with four different LV geometric patterns are presented in Table 1.

Differences between the patients’ groups were found for systolic (sBP) and diastolic (dBP) blood pressure, hemoglobin, IDWG, and LDL cholesterol. Age, gender, body mass index (BMI), HD vintage, serum albumin level, C-reactive protein (CRP), Kt/V were similar between the groups. 

**Figure 2:**
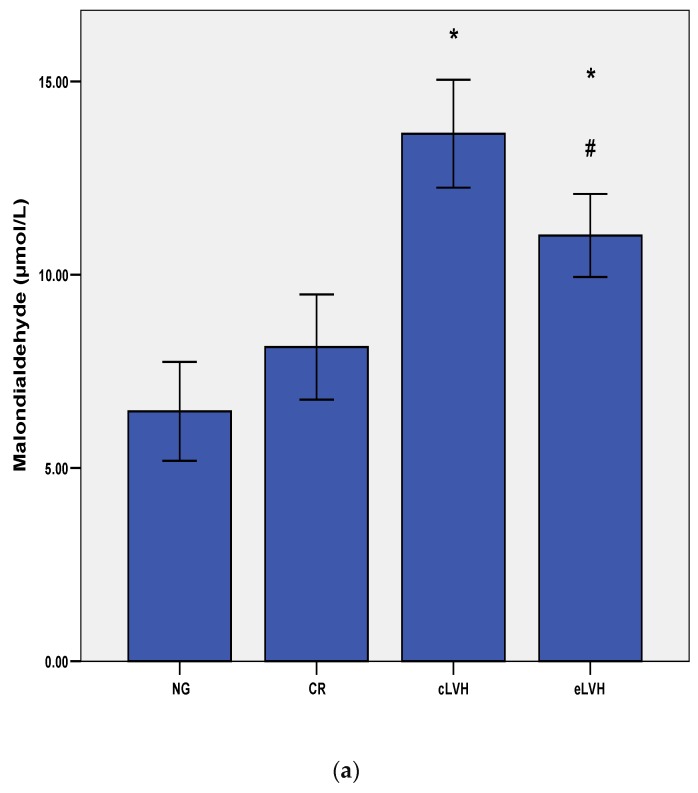

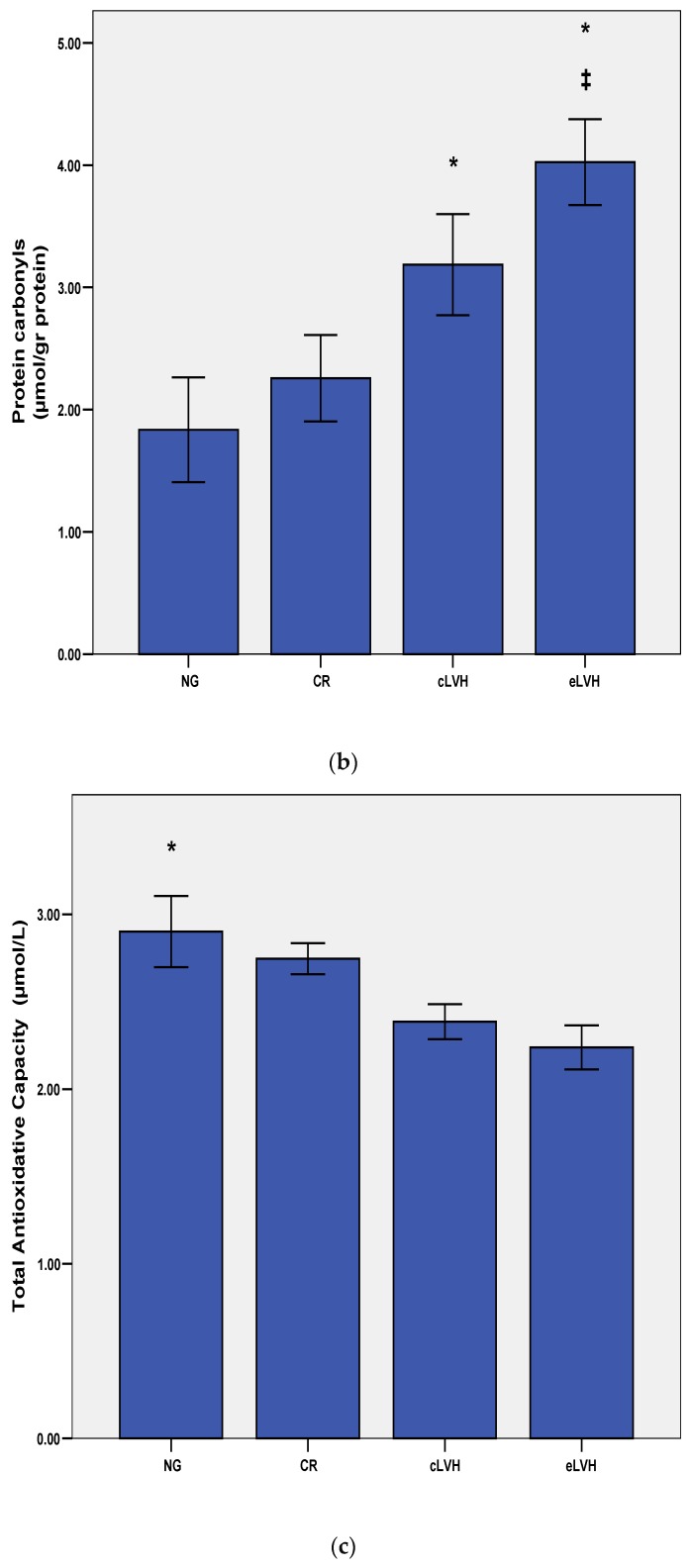
(**a**) Differences in malondialdehyde levels over four groups of patients according to the left ventricular geometry pattern. * *p* < 0.001 compared to NG, # *p* < 0.05 compared to cLVH; (**b**) Differences in protein carbonyl levels over four groups according to the left ventricular geometry pattern. * *p* < 0.001 compared to NG, ‡ *p* = 0.005 compared to cLVH; (**c**) Differences in total antioxidative capacity levels over four groups according to the left ventricular geometry pattern. * *p* < 0.001 compared to CR, cLVH, and eLVH. Abbreviations: NG: normal geometry; CR: concentric remodeling; cLVH: concentric left ventricular hypertrophy; eLVH: eccentric left ventricular hypertrophy.

Patients with left ventricular hypertrophy had elevated oxidative stress measured by MDA, PCs, and TAC, regardless of LVH type. As shown in Figure 2a, mean serum MDA levels in patients with concentric and eccentric hypertrophy were higher than in those with normal geometry (13.64 ± 3.93 and 11.01 ± 3.56 vs. 6.46 ± 2.01 µmol/L respectively, *p* < 0.001). Additionally, we noted a statistically significant difference between MDA levels in cLVH, and eLVH patients (*p* < 0.05).

Similarly, as shown in Figure 2b, serum protein carbonyls levels were significantly higher in patients with cLVH and eLVH compared to patients with the NG (3.18 ± 1.16 and 4.26 ± 1.17 vs. 1.8 ± 0.67 mmol/g of protein, *p* < 0.001, respectively). A significant difference in PC concentration between cLVH and eLVH (*p* = 0.005) was also observed.

As eccentric hypertrophy is largely due to volume overload, we further examined the correlation between the protein carbonyl level and IDWG and found a significant positive correlation (*r* = 0.234, *r* = 0.017).

Additionally, TAC was significantly related to abnormal left ventricular geometry (Figure 2c). In all abnormal LV groups, TAC was a significantly lower (2.23 ± 0.42, 2.38 ± 0.28, and 2.38 ± 0.2 for eLVH, cLVH, and CR group compared to 2.90 ± 0.32 for NG patients, *p* < 0.001).

**Figure 3:**
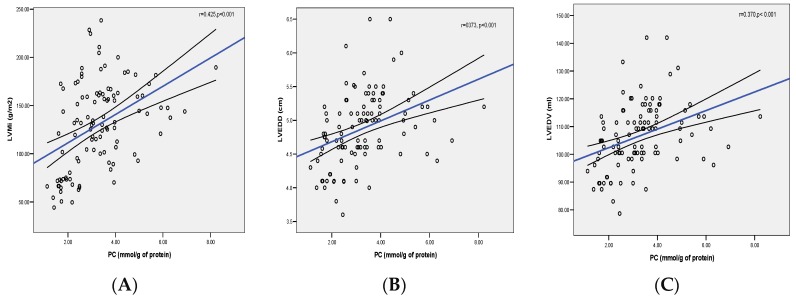
(**A**–**C**) Correlation between PC plasma levels and the indices of eccentric hypertrophy (LVMI, LVEDD, and LVEDV). Abbreviations: PC: protein carbonyl; LVMI: left ventricular mass index; LVEDD: left ventricular end-diastolic diameter; LVEDV: left ventricular end-diastolic volume.

When indices of cardiac remodeling were correlated with the markers of lipid peroxidation, no relations were found between MDA levels and the indices of concentric (LVWT, RWT, and LVMI) nor eccentric hypertrophy (LVEDD and LVEDV). However, protein carbonyls showed a significant positive correlation to LVMI, LVEDD, and LVEDV (*p* < 0.001), as shown in Figure 3A–C, and, to a lesser extent, to LVWT (*p* < 0.05).

Finally, TAC showed a significant, negative correlation with LVMI (*r* = −0.300, *p* = 0.002), LVEDD (*r* = −0.271, *p* = 0.005), LVEDV (*r* = −0.270, *p* = 0.006), in addition to LVWT (*r* = −0.226, *p* = 0.022). TAC did not correlate to RWT.

In unadjusted linear regression analyses, PC significantly correlates with LVEDD and LVEDV (*p* < 0.001) while MDA correlation with these parameters was modest (*p* < 0.05). After multivariable adjustments for covariates, serum protein carbonyls remained statistically significant as a prognostic factor for eccentric LVH type, including greater LVEDD and LVEDV (β = 0.32 and β = 0.28, *p* < 0.001 for both) (Table 2). TAC negatively correlated with both concentric and eccentric LVH phenotypes (*p* < 0.05 in both cases).

As shown in Table 3, the incidence of eccentric LVH increased progressively from the lowest (≤3.49 mmol/g of protein) to intermediate (3.50–5.85 mmol/g of protein) and highest baseline PC tertile (>5.85 mmol/g of protein, *p* < 0.001 for the trend). The predictive role of PCs was anything but marginal, as 2.35 mmol/g of protein of PC increase was associated with a 45% increased risk of eLVH. This negative trend was even more clear when eLVH incidence in the highest PC tertile was compared to that in the lowest tertile, as subjects in the former group exhibited a 76% greater risk of developing eLVH compared to their counterparts. After further adjustment for the potential mediators (Model 2), PCs carried eccentric LVH odds (95% CI) of 1.256 (0.998–1.514), per standard deviation increase. Tertile analysis of the PCs and eccentric LVH showed excess risk with the highest tertile (fully adjusted OR: 1.517, 95% CI: 1.287–1.747).

**Figure 4:**
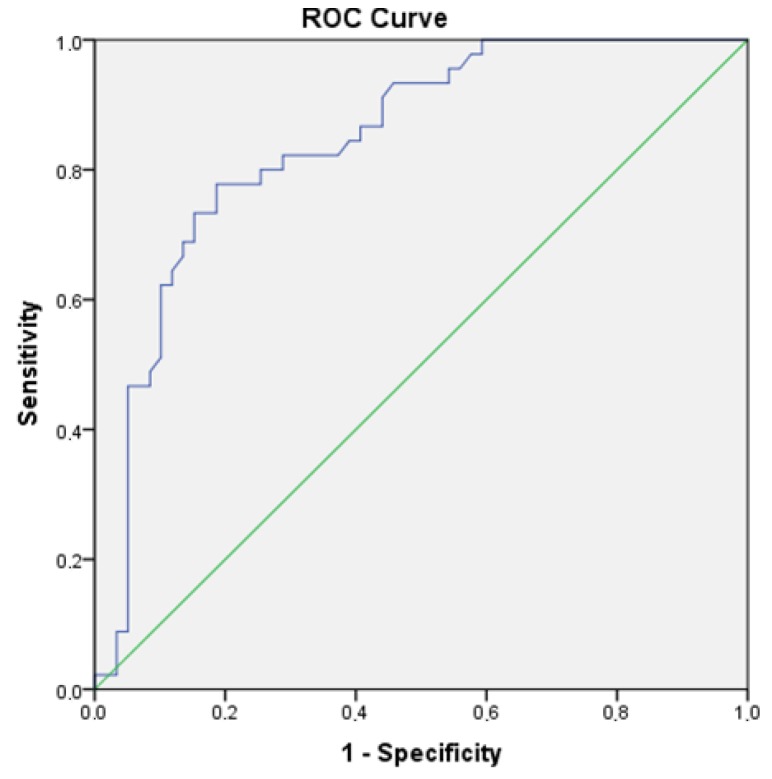
ROC curves for PC concentration in predicting eccentric LVH. Abbreviation: ROC: receiver operating characteristic.

Analysis of the usefulness of plasma PC concentration in the prediction of eccentric LVH yielded an area under the ROC curve of 0.84 (95% confidence interval (CI) 0.77–0.92; *p* < 0.001) (Figure 4). A cutoff of 3.35 mmol/g of protein had a sensitivity of 77.8% and specificity of 81.4%.

## 4. Discussion

We found that end stage renal disease (ESRD) was associated with an alarmingly high prevalence of cardiac remodeling and LVH. In our study, 88.6% of the patients had an abnormal left ventricular chamber, and 75% of them had LVH. These data correlate with the data publicized by several authors [33,34,35]. Most studies [36,37,38], but not all [39], reported that cLVH predominates in non-dialysis-dependent CKD, while the eccentric pattern is the most common in HD patients [40,41]. These abnormal left ventricular geometric patterns have notable prognostic significance and may predict the occurrence of cardiac failure and other cardiovascular diseases.

Emerging data suggest that chronic hemodialysis patients experience heightened oxidative and “carbonyl” stress, which have been proposed as the critical proof linking uremia to cardiovascular complications [42,43,44,45]. Some authors identified redox-dependent changes in cellular proteins and signaling pathways as one of the critical factors involved in left ventricular remodeling and hypertrophy [46,47,48,49].

As the intensification of oxidative stress poses a risk of myocardial mass gain, echocardiographic indices of cardiac remodeling were correlated with biomarkers of oxidative stress.

We showed that oxidative damage of proteins has a significant role in the pathogenesis of eccentric left ventricular hypertrophy. As all four groups of patients had comparable CRP, iron, albumin, and uric acid and all were on bicarbonate dialysis, observed differences between them in terms of oxidative stress and protein carbonyl content could not be linked to varying degrees of inflammation. 

Exposure of proteins to ROS leads to alteration of amino acid residues, protein damage, and an increase in protein carbonyl groups. Massive carbonyl stress affects circulating, cellular, and tissue proteins [13], resulting in their dysfunction. Most protein modifications are irreversible, and oxidative modification in protein structure can generate a broad range of downstream functional consequences as they may cause inhibition of both enzymatic and binding activities and increased susceptibility to aggregation and proteolysis. Association between ROS and indices of ventricular remodeling proves that oxidative damage of proteins plays an influential role in structural changes of the myocardium in hemodialysis patients. Several studies showed that remodeling of cellular organelles from oxidative protein modification might be responsible for the shift from compensatory hypertrophy to ventricular dilation [26,50].

Most of our patients have eccentric hypertrophy, which may indicate poor volume control. As expected, interdialytic weight gain, one of the putative determinants of fluid balance, showed a positive relationship with eLVH. However, the probability of eccentric LVH only modestly increased in the presence of high interdialytic weight gain. After taking into account a number of confounders, protein carbonyls emerged as the most powerful predictor of eccentric LVH.

As previously stated, the presence of protein carbonyl content in patients with eLVH is undoubtedly the consequence of oxidative modifications of proteins. However, based on literature data, it is difficult to conclude which oxidative pathway is responsible for triggering eccentric geometric remodeling of the left ventricle. In many cases, oxidatively modified proteins and advanced glycation end products (AGE) share structural homology; thus, oxidatively modified proteins may serve as candidate ligands for AGE receptors (RAGE) [51].

Conditions with elevated carbonyl stress increase the concentration of RAGE ligand. RAGE signaling activates pathways related to cardiac remodeling [52,53] by activating the TGF-β pathway [54,55,56]. TGF-β induces fibroblast activation and differentiation into myofibroblasts that secrete extracellular matrix proteins and collagen type I, which, in turn, cause eccentric hypertrophy and fibrosis [57].

Finally, the failure to boost the endogenous antioxidant system additionally generated oxidative stress in our study group. Endogenous antioxidants serve as “sacrificial” substances in plasma and extravascular spaces by blocking chain reactions of free radical production. Our study demonstrated that antioxidant defense systems, measured by TAC, were severely affected in patients with abnormal LV geometry probably due to the fact that these systems were overburdened under the state of persistently high levels of ROS in HD patients.

Protein carbonyls have been studied in pediatric patients [58] and for predicting cardiac events in type 2 diabetic patients [59]. In a recent study, carbonyl residues in poorly controlled type 2 diabetes mellitus were positively associated with the cardiovascular risk score [60]. However, to date, only a few published studies evaluated oxidative protein modification products in the light of LVH. Radovanovic et al. found some predictive potentials of protein carbonyls on cardiac remodeling in heart failure patients [61]. To our best knowledge, protein carbonyls had never been studied for the prediction of LVH or cardiac remodeling patterns in end-stage renal disease patients. As we found positive results concerning the role of protein carbonyls in predicting eccentric LVH in HD patients, future studies and clinical trials are needed to confirm the potential clinical importance of these findings.

Our study has certain limitations. First, this is a single-center study with a relatively small sample size; second, we did not compare MDA and PC levels to a non-dialysis population with cLVH and eLVH. We also lack data on diastolic function as we did not perform mitral inflow E- and A-wave velocities. Finally, our findings should be interpreted in light of the cross-sectional design, which limits our ability to assume causation.

## 5. Conclusions

Data on the prognostic potential of biomarkers of oxidative stress on cardiac remodeling are still lacking. The results obtained in this investigation confirmed that oxidative stress parameters might be related to the prognosis of the morphological alterations of LV geometry. High plasma protein carbonyls were associated with increased risk of eccentric LVH in a dose–response fashion. The recognition of the pathophysiological mechanisms which are associated with the cardiac hypertrophy and remodeling process is fundamental for the development of new treatment strategies, primarily because the mortality rates related to cardiac remodeling prevail.

## Figures and Tables

**Table 1:** Demographic, clinical, and echocardiographic characteristics of the study population according to the left ventricular geometry.

Parameters	NG *n* = 12	CR *n* = 14	cLVH *n* = 33	eLVH *n* = 45
Age (years)	51.3 ± 7.4	56.7 ± 24.2	61.1 ± 15.3	60.0 ± 12.7
Gender (f/m)	4/8	4/10	15/18	17/28
HD vintage (months)	52.7 ± 47.3	56.7 ± 55.2	53.5 ± 45.6	57.1 ± 43.8
Body mass index (kg/m^2^)	22.7 ± 1.6	22.1 ± 3.1	23.5 ± 3.6	23.7 ± 3.5
Kt/V	1.35 ± 0.6	1.39 ± 0.8	1.33 ± 0.7	1.36 ± 0.7
Vascular access (AV fistula)	10 (83%)	12 (75%)	25 (78%)	38 (84%)
IDWG (kg)	2.3 ± 1.1	2.8 ± 1.0	2.6 ± 0.9	3.1 ± 0.8 ^a^
sBP (mmHg)	126.7 ± 21.5	135.0 ± 14.0	150.5 ± 15.6 ^a,c^	141.8 ± 20.0 ^d^
dBP (mmHg)	63.6 ± 13.2	65.6 ± 10.0	73.2 ± 7.8 ^d^	72.0 ± 9.5 ^d^
Hemoglobin (g/dL)	11.7 ± 2.0	11.0 ± 1.4	10.3 ± 1.5 ^a,e^	11.2 ± 1.0
Serum albumin (g/dL)	37.3 ± 0.6	34.7 ± 5.1	31.8 ± 5.5	31.7 ± 6.0
CRP (mg/L)	3.3 ± 2.4	3.9 ± 0.7	4.9 ± 0.5	4.1 ± 1.3
Cholesterol (mmol/L)	4.4 ± 0.9	4.16 ± 0.9	4.6 ± 1.3	4.8 ± 1.2
LDL–cholesterol (mmol/L)	1.7 ± 0.2	2.4 ± 0.8	2.7 ± 1.1 ^a^	3.3 ± 0.9 ^a^
HDL–cholesterol (mmol/L)	1.1 ± 0.4	1.2 ± 0.6	1.1 ± 0.1	1.1 ± 0.4
Triglycerides (mmol/L)	2.6 ± 0.9	2.1 ± 1.7	2.0 ± 1.2	2.1 ± 1.3
LVEDD (cm)	4.54 ± 0.36	4.24 ± 0.40	4.86 ± 0.41 ^b,c^	5.17 ± 0.49 ^c,e^
IVST (cm)	0.85 ± 0.17	0.96 ± 0.11	1.34 ± 0.14 ^b,c,e^	1.25 ± 0.13 ^b,c^
PWT (cm)	0.80 ± 0.11	0.92 ± 0.09	1.41 ± 0.16 ^b,c,f^	1.15 ± 0.24.^c^
LVWT (cm)	1.75 ± 0.39	2.12 ± 0.27 ^b^	2.83 ± 0.22 ^b,c,f^	2.46 ± 0.41 ^b,c^
RWT (cm)	0.30 (0.25–0.37)	0.44 (0.43–0.46) ^b^	0.44 (0.43–0.52) ^b,c,f^	0.36 (0.28–0.41) ^b,c^
LVM (g)	121.62 (75.84–188.02)	129.38 (85.96–200.78) ^b^	276.74 (204.79–373.13) ^b,c,f^	249.96 (153.27–340.78) ^b,c^
LVMI (g/m^2^)	67.73 (44.17–101.74)	72.70 (49.60–104.46) ^b^	167.47 (138.50–238.37) ^b,c^	154.07 (130.24–200.07) ^b,c^
LVEDV (mL)	99.42 ± 8.16	92.86 ± 8.85	106.34 ± 8.99 ^f^	113.04 ± 10.84 ^b,c^
LVESV (mL)	37.33 ± 11.06	26.34 ± 5.02 ^a^	43.26 ± 11.01 ^a,b^	37.32 ± 10.84 ^a,b,c^
LVEF (%)	63 (57–70)	57 (51–60) ^b^	60 (55–68) ^b^	61 (57–69)^b^

Data are expressed as mean ± standard deviation or median (interquartile range) and numbers (proportion) as appropriate. CRP: C-reactive protein; IDWG: interdialytic weight gain; NG: normal left ventricle geometry; CR: concentric remodeling; cLVH: concentric left ventricular hypertrophy; eLVH: eccentric left ventricular hypertrophy; HD: hemodialysis; AV fistula: arteriovenous fistula; sBP: systolic blood pressure; dBP: diastolic blood pressure; LVEDD: left ventricular end-diastolic diameter; IVST: interventricular septal thickness; PWT: posterior wall thickness; LVWT: LV wall thickness; RWT: relative wall thickness; LVM: left ventricular mass; LVMI: left ventricular mass index; LVEDV: left ventricular end-diastolic volume; LVESV: left ventricular end-systolic volume; LVEF: left ventricular ejection fraction; LVH: left ventricular hypertrophy. ^a^ Statistically significant difference compared to NG group of patients (*p* < 0.05); ^b^ Statistically significant difference compared to NG group of patients (*p* < 0.001); ^c^ Statistically significant difference compared to CR group of patients (*p* < 0.001); ^d^ Statistically significant difference compared to CR group of patients (*p* < 0.05); ^e^ Statistically significant difference compared to group eLVH group of patients (*p* < 0.05); ^f^ Statistically significant difference compared to eLVH group of patients (*p* < 0.001).

**Table 2:** Multiple regression models of LVMI, LVWT, RWT, LVEDD, and LVEDV.

	β	*p*
**Dependent variable: LVMI ^a^*R*^2^ = 0.57; *p* < 0.001**		
**Independent variable**		
MDA	0.266	0.011
PC	0.328	<0.001
TAC	−0.177	0.043
HD vintage	0.231	0.010
Hemoglobin	−0.337	<0.001
**Dependent variable: LVWT ^b^*R*^2^ = 0.61; *p* < 0.001**		
**Independent variable**		
PC	0.288	0.013
TAC	0.266	0.011
MDA	0.038	0.22
Hemoglobin	−0.31	0.002
**Dependent variable: RWT ^c^ R^2^ = 0.53; *p* < 0.001**		
**Independent variable**		
IDWG	−0.22	0.002
MDA	0.04	0.072
PC	0.19	0.003
Hgb	−0.33	0.002
sBP	0.27	0.001
**Dependent variable: LVEDD ^d^*R*^2^ = 0.50; *p* < 0.001**		
**Independent variable**		
PC	0.32	<0.001
MDA	0.16	0.06
TAC	0.18	0.008
IDWG	−0.17	0.01
**Dependent variable: LVEDV ^e^*R*^2^ = 0.48; *p* < 0.001**		
**Independent variable**		
*PC*	0.28	<0.001
TAC	0.12	0.04
IDWG	−0.10	0.02

Data are expressed as standardized regression coefficients (b) and *p* values. ^a^ Out of the model: IDWG, LDL—cholesterol, gender, sBP, dPB, age, albumin, calcium; ^b^ Out of the model: LDL—cholesterol, gender, IDWG, HD vintage, sBP, dPB, age, albumin, calcium; ^c^ Out of the model: LDL—cholesterol, TAC, gender, HD vintage, dPB, age, albumin, calcium, kt/V; ^d^ Out of the model: LDL—cholesterol, gender, HD vintage, sBP, dPB, age, albumin, calcium, Hgb; ^e^ Out of the model: LDL—cholesterol, MDA, gender, HD vintage, sBP, dPB, age, albumins, calcium, Hgb.

**Table 3:** Odds ratio of two different LVH patterns according to continuous or tertiles of PCs.

Variables	Eccentric LVH		Concentric LVH	
	Model 1	Model 2	Model 1	Model 2
PC (Per 1 SD increase)	1.344 (1.203–1.503 ^a^	1.256 (0.998–1.514) ^a^	1.321(1.285–1.408) ^a^	1.094 (0.875–2.181) ^c^
Tertile of PC				
T1 (≤3.49)	1.000 (reference)	1.000 (reference)	1.000 (reference)	1.000 (reference)
T2 (3.50–5.85)	1.446 (1.277–1.615) ^a^	1.366 (1.218–1.533) ^a^	1.421 (1.277–1603) ^a^	1.248 (1.180–1.390) ^c^
T3 (>5.85)	1.766 (1.510–2.029) ^a^	1.517 (1.287–1.747) ^a^	1688 (1.492–1884) ^a^	1.344 (1.211–1.507) ^c^
*p* for trend	<0.001	<0.001	<0.05	0.04

Abbreviations: OR: odds ratio; MDA: malondialdehyde; PC: protein carbonyl; SD: standard deviation. Model 1: unadjusted; Model 2: adjusted for LDL—cholesterol, gender, HD vintage, sBP, dPB, age, albumins, calcium, Hgb, IDWG, TAC. ^a^ *p* < 0.001, ^c^ *p* < 0.05.
